# Palpitation post-vaccination: What should we do?

**DOI:** 10.51866/tyk.935

**Published:** 2025-11-26

**Authors:** Samat Farhani, Aidi Zaharudin, Nur Athirah Rosli, Jue Jing Ong, T. Ramanathan Sharmilee, Siti Athirah Baharuddin

**Affiliations:** 1 MD, Master in Medicine (Fam Med), Klinik Kesihatan Tanjong Karang, Jalan Lengkungan, Tanjong Karang, Selangor, Malaysia. Email: hani5204@gmail.com; 2 MD, MAFP/icFRACGP, Klinik Kesihatan Kalumpang Kalumpang, Kerling, Selangor, Malaysia.; 3 MBBS, Master in Medicine (Fam Med), Klinik Kesihatan Tanjong Karang Jalan Lengkungan, Tanjong Karang, Selangor, Malaysia.; 4 MBBS, MAFP/FRACGP, Klinik Kesihatan Kuala Selangor Jalan Klinik, Taman Malawati, Kuala Selangor, Selangor, Malaysia.; 5 MBBS, MAFP/FRACGP, Klinik Kesihatan Puchong Batu 141, Jalan PU 5, Taman Puchong Utama, Puchong, Selangor, Malaysia.; 6 MBBS, MAFP/icFRACGP, Klinik Kesihatan Tanjong Karang Jalan Lengkungan, Tanjong Karang, Selangor, Malaysia.

**Keywords:** Myocarditis, Vaccines, COVID-19

## Abstract

In this clinical dilemma, we highlight the case of a previously healthy 37-year-old lady who presented to a primary care clinic with palpitations, chest discomfort and difficulty breathing within 2 hours post-mRNA COVID-19 vaccine injection. During the initial presentation to the clinic and subsequent admission to the ward, there were no significant findings except for intermittent palpitations, which were relieved by supine positioning. Serial electrocardiograms and blood investigations, including troponin-I and thyroid function tests, revealed normal findings. Given persistent symptoms, further referrals were made. She underwent a tilt table test, which demonstrated normal results. Cardiac MRI showed myocardial oedema and inflammation at the basal anteroseptal wall. Herein, we discuss several differential diagnoses, clinical features and appropriate management for this patient. This case highlights the importance of having a high clinical index of suspicion and involving a multidisciplinary team in managing a case with a diagnostic dilemma.

## Case summary

A 37-year-old woman with no known medical illness presented to a primary care clinic with palpitations, chest discomfort and shortness of breath 2 h following her second dose of the Pfizer-BioNTech COVID-19 vaccine. On evaluation, she appeared unwell and tachycardic (heart rate [HR]: 110 beats per minute [bpm]). Her symptoms worsened upon standing and were relieved by supine positioning. She was admitted for further evaluation of adverse events following vaccination.

**Figure 1:**
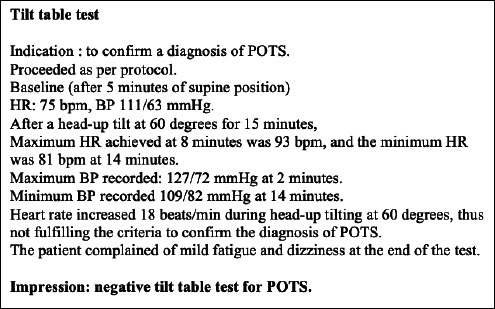
Tilttable test report summary.

**Figure 2:**
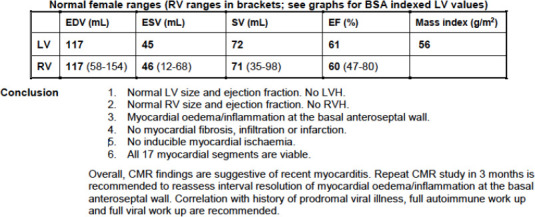
Cardiac MRI report.

Physical examination findings were unremarkable. Bedside examination revealed no orthostatic hypotension. Initial investigations, including serial electrocardiograms (ECGs), serial troponin-I tests, thyroid function test, renal profile, full blood count, liver function test and electrolyte assessment, all yielded normal findings. Transthoracic echocardiogram showed a preserved left ventricular ejection fraction (LVEF) of 68% with normal ventricular function. She was discharged with T. bisoprolol once daily. Further referrals to the cardiology and neurology teams were made. Figure 1 depicts the tilt table test report summary, while Table 1 displays the Holter report summary a month after the presentation of symptoms. Cardiac MRI arranged by the cardiology team subsequently showed myocardial oedema and inflammation at the basal anteroseptal wall (Figure 2).

**Table 1:** Holter report summary (1 month after initié presentation).

Day	Heart rate (beat per minute)	Ventricular ectopic	Patient diary event	Event outcome
1	72	2 ectopics	5 times	Sinus rhythm, rate: 58-92
2	65	2 ectopics	2 times	Sinus rhythm, rate: 62-80
3	70	1 ectopic	2 times	Sinusrhythm, rate: 99–137

**Questions:**

1. What is the most likely differential diagnosis?
2. What further investigation can be done?
3. What are the pathophysiology and complications that may arise from this condition?

**Answers with discussion**

1. The most likely differential diagnosis is post-vaccination myocarditis and postural orthostatic tachycardia syndrome (POTS). The presentation of post-vaccination myocarditis typically starts within a few days of COVID-19 vaccination and most commonly occurs after the second dose of vaccine. The presentation can be mild and non-specific. Symptoms reported include chest pain, dyspnoea and dizziness.^1^ The European Society of Cardiology (ESC) defines clinical myocarditis as having at least three of the following symptoms: chest pain, shortness of breath or palpitations; and at least three different types of diagnostic criteria, such as electrocardiographic features of cardiac injury, elevated markers of myocardial necrosis and functional or structural abnormalities on cardiac MRI, echocardiogram or angiogram without coronary stenosis (>50%) and known pre-existing heart disease. With POTS, the symptoms can vary between individuals but often include light-headedness and palpitations (as in this case), along with generalised weakness and blurred vision.^2^ These symptoms are characterised by orthostatic intolerance that is worse while upright, with rapid improvement upon return to supine positioning. The diagnosis of POTS must include these characteristics as well as the following:
  a) Sustained HR increase of ≥30 bpm (or ≥40 bpm if patient is aged 12-19 years) within 10 min of upright posture (tilt table test)
  b) Absence of significant orthostatic hypotension (magnitude of blood pressure drop of ≥20/10 mmHg).
  It is challenging to distinguish between the two diagnoses in this case presentation before performing the tilt table test. The test indicated that the patient’s HR increased by 18 bpm during head-up tilting at 60°; hence, a POTS diagnosis was excluded.
2. Cardiac MRI
  The diagnosis of myocarditis in this case is also challenging. Initially, computed tomography pulmonary angiogram (CTPA) was planned for the patient. Upon the CTPA appointment, the patient developed chest discomfort associated with shortness of breath and palpitations. The ECG at that time showed sinus rhythm with T inversion at III and a partial right bundle branch block. Chest radiography showed some mild thickening at the top of the right pleura, while echocardiogram revealed that the LVEF was 54%, and the valves were normal in shape. CTPA showed no evidence of pulmonary embolism. She then underwent computed tomography coronary angiogram (CTCA), which demonstrated a possible non-calcified plaque at the midsegment with mild (30%—40%) stenosis at the right coronary artery. These findings led to her diagnosis of mild coronary artery disease involving the right middle coronary artery.
  Further evaluations were conducted with cardiac MRI by the cardiology team, and the reports showed the presence of myocardial oedema and inflammation at the basal anteroseptal wall (Figure 2). The cardiac MRI findings were suggestive of myocarditis. In this case, diagnosing myocarditis in the absence of elevated cardiac biomarkers such as troponin is challenging, but a multifaceted approach combining clinical suspicion, other laboratory tests and advanced imaging is essential. According to the ESC guidelines, clinical myocarditis is diagnosed when a patient shows symptoms such as chest pain, shortness of breath or heart palpitations, along with certain test results that indicate heart damage, such as changes seen on an ECG, high levels of heart injury markers or issues found on cardiac MRI, echocardiogram or angiogram, as long as there is no significant narrowing of the coronary arteries (>50%) or any known heart disease. Cardiac MRI is considered the gold-standard non-invasive tool for diagnosing myocarditis.^3^ It can detect myocardial oedema, inflammation and injury even when biomarkers are normal. While endomyocardial biopsy remains the definitive diagnostic standard, it is an invasive procedure.^4^ It is typically reserved for severe or fulminant cases where the diagnosis is unclear or the patient is not responding to therapy. Thus, a stepped approach is recommended for a family physician after a comprehensive evaluation that includes thorough history-taking, physical examination and initial screening tests and discussion with the cardiologist, as the widespread cardiac MRI approach for this symptom alone would be impractical and costly and could lead to unnecessary anxiety and further investigations.^5^
3. Myocarditis is a polymorphic, underdiagnosed disease with a range of clinical manifestations, courses and prognoses. There are numerous factors that can induce myocarditis, including infections (e.g. viruses, bacteria or parasites), autoimmune disorders, hypersensitivity, high catecholamine states, medications, toxic compounds or physical agents. Once other particular causes have been ruled out, immunological responses and viral infections account for the majority of myocarditis cases seen in clinical practice. The incidence rate of post-vaccination myocarditis worldwide ranges from 0.58 to 15.07 per 100,000 individuals.^1^ Male sex is predominant in most studies, but one population-based study in Denmark recorded female sex predominance.^2^ The occurrence of myocarditis post-vaccination has been associated with age of 12-39 years. The pathophysiology of COVID-19 vaccine-associated myocarditis remains unknown, and it is difficult to ascertain this association. Danish studies showed that the link between myocarditis and COVID-19 mRNA vaccination was weak, with rates of 1.3 (0.8-1.9) cases per 100,000 people within 28 days of vaccination. Some case reports showed changes on ECG (e.g. ST depression) along with a troponin-I level (6.14 ng/mL) that was much higher than the normal range of 0-0.30.^1^
  The presentation of myocarditis postvaccination typically starts within a few days of the COVID-19 vaccination and most commonly occurs after the second dose of the vaccine. The presentation can be mild and non-specific. Symptoms reported include chest pain, dyspnoea and dizziness.^6^ The disease can progress to fulminant myocarditis, cardiomyopathy and mortality if left untreated. Longterm sequelae vary, such as systolic dysfunction, residual left ventricular dysfunction and sudden cardiac death. Treatment for myocarditis ranges from corticosteroids or immunosuppressive therapy to intravenous immunoglobulin to reduce inflammation.^6,7^ Therefore, medical therapy should be started following specific complications during follow-up. Continuous surveillance and monitoring of patients with prevention exercise after ^3^ months following diagnosis are suggested.^7^ Cardiac MRI abnormalities consistent with myocarditis associated with mRNA vaccine administration may improve over the subsequent several months, but evidence of necrosis or fibrosis via late gadolinium enhancement may not completely resolve, suggesting the presence of residual fibrosis.^8^ This scar tissue can become an area of electrical instability, leading to abnormal heart rhythms that patients perceive as palpitations, as in this case report.

## Case progress

The diagnosis was made 4 months after the vaccination. During her visits to the cardiologist, she was started on T. bisoprolol 2.5 mg once daily, T. clopidogrel 75 mg once daily and T. atorvastatin 10 mg at night. Her repeat cardiac MRI a year later showed resolution of myocardial oedema. However, because of the persistent symptoms of intermittent palpitations that the patient had, she continued to be on beta-blocker and statin therapy. A cardiologist and primary care physician are currently monitoring her illness.
